# Esoteric Beliefs and Opposition to Corona Restrictions in Germany

**DOI:** 10.1007/s11615-023-00468-0

**Published:** 2023-05-30

**Authors:** Nils B. Weidmann

**Affiliations:** Department of Politics and Public Administration, University of Konstanz, 78457 Konstanz, Germany

**Keywords:** Esoteric beliefs, Homeopathy, Corona measures, Spatial analysis, Esoterische Überzeugungen, Homöopathie, Corona-Maßnahmen, Räumliche Analyse

## Abstract

Governmental measures against the spread of the Corona virus have been met with varying levels of opposition in many countries worldwide. Existing research has claimed that some of this opposition is linked to esoteric and anthroposophical beliefs. This research note tests this in an observational study using election results from the 2021 parliamentary election in Germany and new data on the distribution of natural healers, homeopathic doctors and Steiner schools. Results show that counter to common expectations, there is no evidence that esoteric beliefs systematically lead to increased support for the established right-wing *AFD*. Rather, some indicators for esoteric beliefs – in particular, the presence of homeopathic doctors and Waldorf schools – are related to higher support for the new fringe party *dieBasis*, a single-issue party campaigning against governmental Corona measures.

## Abstract (de)

Staatliche Maßnahmen gegen die Ausbreitung des Corona-Virus stießen in vielen Ländern weltweit auf unterschiedlichen Widerstand. Die bisherige Forschung vermutete, dass ein Teil dieser Opposition auf esoterische und anthroposophische Überzeugungen zurückzuführen ist. Der vorliegende Forschungsbeitrag untersucht diesen Zusammenhang in einer Beobachtungsstudie anhand von Wahlergebnissen der Bundestagswahl 2021 in Deutschland und mit neuen Daten zur Verteilung von Heilpraktikern, homöopathischen Ärzten und Waldorfschulen. Die Ergebnisse zeigen, dass es entgegen allgemeiner Erwartungen keine Hinweise darauf gibt, dass esoterische Überzeugungen systematisch zu einer verstärkten Unterstützung der etablierten rechten *AFD* führen. Vielmehr zeigen einige Indikatoren für esoterische Überzeugungen – insbesondere die Präsenz homöopathischer Ärzte und Waldorfschulen – einen Zusammenhang mit einem erhöhten Stimmenanteil für die neue Randpartei *dieBasis*, die sich speziell gegen staatliche Corona-Maßnahmen einsetzte.

To limit the spread of the Coronavirus, governments worldwide have imposed, sometimes drastic, measures on citizens. Across many countries, restrictions of basic rights and freedoms were put in place and have had major impacts on the lives of many. Not surprisingly, these measures were heavily contested, and the varying levels of acceptance of Corona restrictions across different societal groups have become an important area of research. What characteristics determine whether people oppose face mask mandates or Covid-19 vaccinations, or deny the severity of the disease altogether?

For several European countries, observers have claimed that rejection of Covid restrictions is related to esoteric beliefs and support of alternative healing methods. Some research, indeed, points in this direction. For example, spirituality emerged as one consistent predictor of Covid-related conspiratorial beliefs (Gligorić et al. 2021). Other work suggests that public demonstrations against Corona measures were, indeed, attended by disproportionate numbers of people with esoteric and anthroposophical backgrounds (Frei and Nachtwey 2022). Yet, it remains unclear whether this result holds more generally, and whether these beliefs actually translate into manifest political behavior.

This study aims to test the relationship between esoteric beliefs and opposition against governmental measures against Corona in an observational, comparative study of Germany. Germany is a particularly interesting case, since certain esoteric forms, such as homeopathy and anthroposophy, are widespread. For example, according to a recent survey, homeopathic treatments are used by up to 60% of the population, and a majority believes that they are at least partially effective (Hartmann 2023). The country hosts the largest number of Steiner schools worldwide (Paull and Hennig 2020), which, while run by non-state organizations, are licensed as part of Germany’s education system. In addition, Germany features a great number of natural healers practicing alternative medicine, for which there is a high demand in the population (Bund Deutscher Heilpraktiker [German Association of Natural Healers] 2017). Common to all these belief systems is that they rely on “powers” of different kinds, such as, for example, natural “energies,” which do not hold up to systematic empirical observation and lack support according to commonly accepted standards of scientific evidence.

Specifically, the study asks (i) whether these beliefs are systematically linked to political support for political parties explicitly opposing these measures, and (ii) which of these parties is able to benefit from them. The latter question is important, since opposition against Corona measures is usually associated with the political right. For example, research has shown that adherence to social distancing rules in Italy was significantly lower in provinces with higher support for right-wing parties (Barbieri and Bonini 2021). A survey conducted by the Pew Research Center also confirms that opposition to Corona restrictions in some of the most advanced economies worldwide can primarily be found on the ideological right (Connaughton 2021). However, this narrative may be too simple. Opposition against Corona restrictions based on esoteric beliefs is likely to originate also from social groups that are less likely to support the traditional extreme right.

This is why the analysis below tests the relationship between esoteric beliefs and political support for two parties that in the 2021 parliamentary election explicitly opposed the governmental measures against Corona. Most importantly, this was the right-wing *Alternative für Deutschland* [Alternative for Germany, *AFD*], which quickly adjusted to the new issue and was crucial in organizing much of the anti-government mobilization that occurred in Germany (Lehmann and Zehnter 2022). However, the election also gave rise to a new party, the *Basisdemokratische Partei Deutschland* (*dieBasis* [The Base]). The party grew out of Germany’s main anti-Corona protest movement and ran entirely on its critical stance towards governmental Corona measures (Schmitz-Vardar 2021).

The analysis uses new indicators for esoteric beliefs at a fine-grained spatial resolution: the location of natural healers, homeopathic doctors and Steiner (“Waldorf”) schools. It tests how these indicators are related to electoral support for *dieBasis* and the *AFD*, using analyses at the level of electoral districts and municipalities. The latter approach makes it possible to create matched samples to improve causal inference. The results show that dieBasis seemed to benefit considerably from esoteric beliefs in the population; their vote share is significantly higher in areas with more homeopathic doctors and with Steiner schools. For the *AFD*, there is no such relationship. If anything, support for the *AFD* is lower in these areas. Hence, the analysis shows that from esoteric circles, a new type of anti-governmental sentiment emerged in Germany, which cannot be attributed to the established political right. At the same time, however, the magnitude of this sentiment in the population is much lower, as is shown by the low vote shares of *dieBasis*.

## Research Design and Data

Regression analysis with spatial data is used to test the relationship between the presence of esoteric beliefs and opposition to Corona measures, the latter measured both as support for *dieBasis* and the *AFD* in the German parliamentary election in September 2021. The analysis uses two different levels of resolution: (i) electoral districts and (ii) municipalities.

### District Level

Data on election results at the electoral district level ($N=299$) was obtained from the German electoral commission (Bundeswahlleiter [German Federal Electoral Commission] 2021a). In Germany’s electoral system, voters have two votes, one for a constituency-level candidate, and one for a party list. To rule out candidate effects, I use the second vote for all election results in the analysis below. *dieBasis* received 1.4% of the (second) votes at the national level, with district-level results ranging from 0.6% to 3.5% ($\text{sd}=0.48$). While this is not much compared to the established parties, it was not a bad result for the first national election that the party participated in, making it the third-largest party among those below the 5% electoral threshold. The AFD received 10.3% of the second votes, and district-level results ranged from 2.9% to 33.5% ($\text{sd}=5.9$).

To gauge the prevalence of esoteric beliefs, I use data on the location of natural healers, homeopathic doctors and Steiner (“Waldorf”) schools. Natural healers and homeopathic doctors offer treatments in alternative medicine, relying on methods that have no scientific basis and are not proven to be effective in medical studies. Since medical services are overwhelmingly rendered to patients in person and not remotely, the location of a healer or a doctor is assumed to indicate that a significant population of patients exists in close proximity. For the indicator based on Steiner schools, more elaboration is required. In the German federal system, public education is one of the responsibilities of the state governments, which also decide about the location of public schools. Steiner schools, in contrast, are schools outside the governmental system. While receiving most of their funding from the state once they have been certified, they are founded in a bottom-up process based on the initiative of local associations. Hence, the presence of a local Waldorf school is indicative of a significant number of people supporting Steiner’s anthroposophical ideology.

**Fig. 1:**
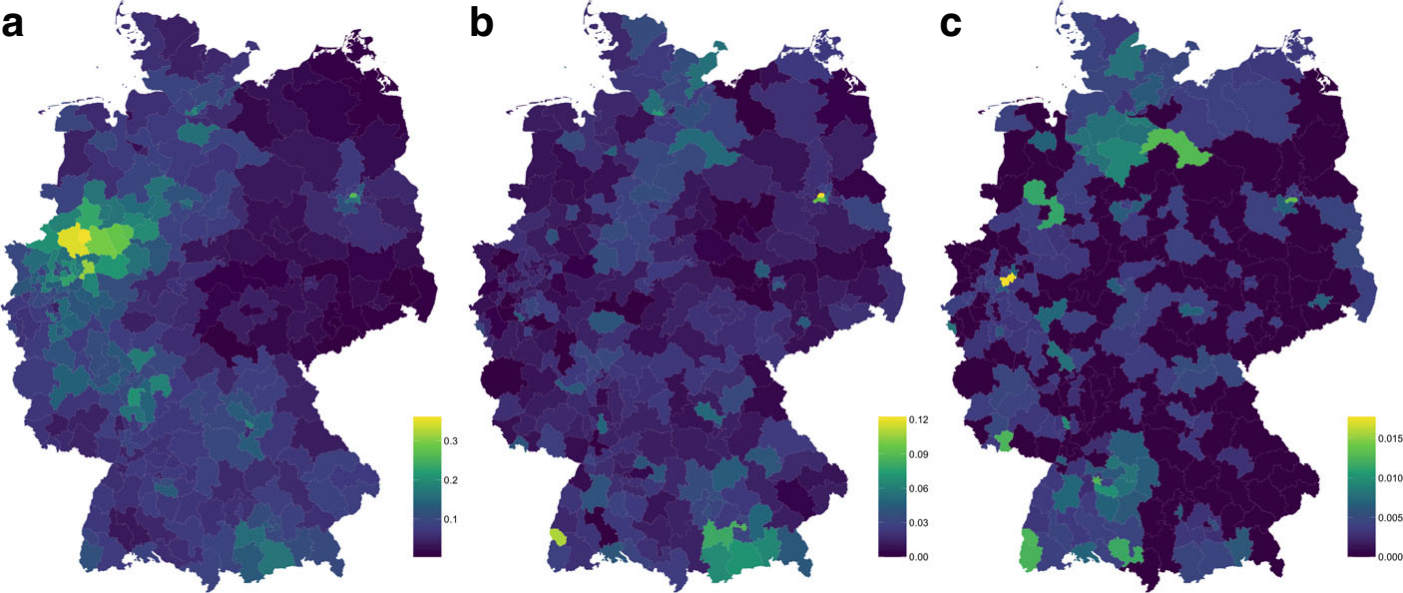
Rate of natural healers (**a**), homeopathic doctors (**b**) and Steiner schools (**c**) across Germany, per 1,000 population

The data for the analysis below was generated from publicly available information. The main German association of natural healers, the “Bund Deutscher Heilpraktiker”, maintains a public directory of healers in Germany with 7751 entries.^1^ This directory was converted to tabular format and only the street addresses were retained. A similar approach was used to obtain data on the distribution of homeopathic doctors across Germany. Doctors are registered with Germany’s professional organization for homeopathic doctors, the “Deutscher Zentralverein homöopathischer Ärzte” (DZVhÄ). The public directory contains 1790 entries with complete addresses of doctors.^2^ Finally, the analysis uses data from Germany’s association of free Waldorf schools (“Bund Freier Waldorfschulen”, BFW), which publishes a freely accessible list of schools in Germany.^3^ From this list, only the schools were extracted, and other facilities such as teacher training or convention centers were excluded. The addresses of healers, doctors and schools were geo-referenced using the Google Maps API to yield the geographic coordinates (longitude/latitude), such that they could be joined with a GIS dataset on district boundaries according to their location. This way, we obtained a count of healers, doctors and schools per district. Fig. 1 maps these counts relative to the district population.

Healers are distributed unevenly across Germany. While the average number of healers per district is about 26, some districts have more than 100. If measured relative to the population, most of the districts with a high per capita rate of healers are located in the state of North-Rhine Westphalia (see the map in Fig. 1a). Districts also exhibit great variation in the numbers of homeopathic doctors. Several have none, while others have more than 30 doctors. Relative to population, cities in the south such as Freiburg and Munich have the highest rates of homeopathic doctors, but so do also other major urban centers such as Berlin or Hamburg. Fig. 1b displays the varying density of doctors across the country, showing the large concentration in the south. For Steiner schools, the city of Stuttgart appears close to the top of the list, since this is where the world’s first Steiner school was founded in 1919. Still, other districts also host several schools, such as the Ennepe-Ruhr district II (with a total of four schools in Witten and Benefeld), Freiburg in the south-west or Berlin. The map in Fig. 1c shows the distribution across Germany.

### Municipality Level

**Fig. 2:**
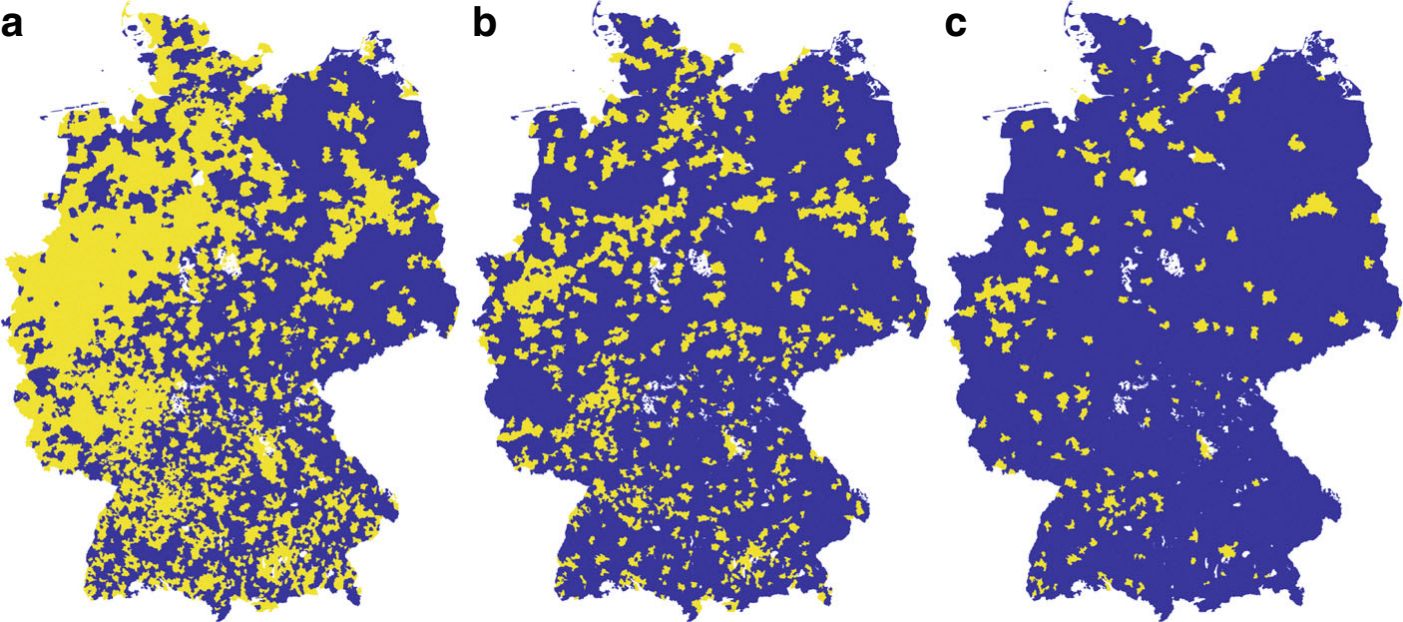
Presence (0/1) of at least one natural healer (**a**), homeopathic doctor (**b**) and Steiner school (**c**) across municipalities in Germany

For the analysis below, another dataset at a finer resolution (municipalities) was created. Municipality borders were obtained from the German Federal Agency for Cartography and Geodesy (2022). Election results were obtained from the German electoral commission (2021a) at the level of municipal electoral districts ($N=94,668$) and aggregated to the level of municipalities. In many cases, several municipalities jointly count their mail-in ballots; these municipalities were combined and treated as a single one to properly attribute postal votes (which were influential in the 2021 election, due to the pandemic situation). After this aggregation, the final municipality dataset has 6,186 entries. Fig. 2 shows the distribution of healers, doctors and schools at the municipality level.

The three maps in Fig. 2 show that, unsurprisingly, healers are much more widespread in Germany than doctors and schools. However, the maps also indicate differences between East and West Germany, with esoteric beliefs – as measured by the three indicators – being much more widespread in the west. This can be recognized most clearly for natural healers, where the healer density is markedly lower in the five states that formerly constituted the German Democratic Republic (Fig. 2a). Similar, although less pronounced, differences can be observed for homeopathic doctors (Fig. 2b) and Steiner schools (Fig. 2c). This is to a large extent a historic legacy; schools outside the state-based educational system such as Waldorf schools were not permitted in the GDR. Homeopathic doctors and natural healers were barely tolerated, and their numbers remained very low. The analysis below eliminates these different legacies in the East and the West by focusing entirely on variation within the federal states.

## Results

The following sections present empirical results on the relationship between esoteric beliefs and electoral support for *dieBasis* and the *AFD*, starting with the district level analysis before moving on to the municipality level.

### District Level

In the subsequent analysis, we use regression models with the electoral district vote share for *dieBasis* and the *AFD* as dependent variable. The models include different structural variables as controls, such as the degree of urbanization, the average household income, and the unemployment rate. These variables were obtained from an associated dataset with structural data for the electoral districts, created by the electoral commission (2021b). All data are for the year 2020, which is the last available year in the collection. In addition, the analysis includes an indicator for urban districts, which takes the value of 1 if a district contains a major city with a population of 80,000 or above (or if the district belongs to one of the city states Berlin, Hamburg and Bremen). The location of these cities was obtained from SimpleMaps (2022). The district level models are OLS regressions with state level fixed effects and clustered standard errors at the state level. This nets out systematic differences between states, for example those between states in the East and the West. The predictor variables on the presence of esoteric beliefs are included as densities (per 1,000 population), to account for the different sizes of electoral districts.

**Table 1:** OLS regression results at the electoral district level. Dependent variable: *dieBasis* vote share. Standard errors clustered at the state level

	*Dependent variable:*					
	*dieBasis* vote share					
	(1)	(2)	(3)	(4)	(5)	(6)
Healer rate	$-$0.263				$-$0.712	$-$0.752
	(0.706)				(0.617)	(0.629)
Doctor rate		4.448${}^{*}$			4.001${}^{*}$	4.213${}^{*}$
		(2.078)			(2.000)	(1.981)
School rate			22.682$${}^{***}$$		19.801$${}^{***}$$	
			(4.191)		(3.089)	
School (0/1)				0.141$${}^{***}$$		0.130$${}^{**}$$
				(0.047)		(0.048)
Urban	$-$0.078	$-$0.111	$-$0.111${}^{*}$	$-$0.122${}^{*}$	$-$0.126${}^{*}$	$-$0.139${}^{*}$
	(0.058)	(0.069)	(0.058)	(0.066)	(0.069)	(0.078)
Income	0.008	$-$0.002	0.002	$-$0.00000	$-$0.003	$-$0.005
	(0.011)	(0.017)	(0.010)	(0.012)	(0.016)	(0.018)
Unemployment	$$-$$0.036$${}^{**}$$	$$-$$0.040$${}^{*}$$	$$-$$0.037$${}^{*}$$	$$-$$0.038$${}^{*}$$	$$-$$0.044$${}^{**}$$	$$-$$0.046$${}^{**}$$
	(0.015)	(0.021)	(0.018)	(0.019)	(0.019)	(0.020)
State FEs	Yes	Yes	Yes	Yes	Yes	Yes
Clustered SEs (state)	Yes	Yes	Yes	Yes	Yes	Yes
Observations	287	287	287	287	287	287
Adjusted R${}^{2}$	0.504	0.520	0.521	0.521	0.531	0.532

*Note:* $${}^{*}$$ $$p<0.1$$; $${}^{**}$$ $$p<0.05$$; $${}^{***}$$ $$p<0.01$$

The first set of models uses the vote share for *dieBasis* as the dependent variable. Models 1–3 in Table 1 present regression results using each of the three indicators for esoteric beliefs individually. The density of natural healers has no discernible effect on the vote share for *dieBasis*, while for homeopathic doctors and Steiner schools, the correlation is positive. Model 4 in Table 1 uses an alternative binary indicator (0/1) for the presence of a school, which receives a positive and significant coefficient. Models 5 and 6 show that the effects for doctors and schools persist in direction and magnitude when all three independent variables are included jointly, which shows that the indicators have little overlap and measure different types of beliefs. Increasing the rate of doctors from the 5th to the 95th percentile in Model 5, the vote share for *dieBasis* increases by about 0.19 percentage points, while the same increase in the rate of Steiner schools is related to an increased vote share by 0.16 percentage points. In Model 6, the effect of doctors is similar to the previous model, while the effect of Steiner schools is easier to interpret; here, a district with a school is predicted to have a vote share for dieBasis that 0.13 percentage points higher than for a district without a school. The estimated coefficients for the control variables show that resistance to Corona measures is higher in more rural areas and negatively correlated with unemployment.

**Table 2:** OLS regression results at the electoral district level. Dependent variable: *AFD* vote share. Standard errors clustered at the state level

	*Dependent variable:*					
	*AFD* vote share					
	(1)	(2)	(3)	(4)	(5)	(6)
Healer rate	$$-$$17.759$${}^{**}$$				$$-$$12.194$${}^{*}$$	$$-$$11.656$${}^{*}$$
	(8.029)				(6.135)	(6.074)
Doctor rate		$$-$$56.350$${}^{***}$$			$$-$$40.220$${}^{***}$$	$$-$$41.090$${}^{***}$$
		(12.411)			(11.464)	(10.556)
School rate			$$-$$202.616$${}^{***}$$		$$-$$115.552$${}^{**}$$	
			(59.976)		(40.530)	
School (0/1)				$$-$$1.390$${}^{***}$$		$$-$$0.932$${}^{**}$$
				(0.427)		(0.317)
Urban	$$-$$1.581$${}^{**}$$	$$-$$1.406$${}^{**}$$	$$-$$1.521$${}^{**}$$	$$-$$1.388$${}^{**}$$	$$-$$1.224$${}^{*}$$	$$-$$1.108$${}^{*}$$
	(0.621)	(0.622)	(0.657)	(0.597)	(0.610)	(0.562)
Income	$$-$$0.303$${}^{**}$$	$$-$$0.258$${}^{**}$$	$$-$$0.328$${}^{**}$$	$$-$$0.308$${}^{**}$$	$$-$$0.215$${}^{*}$$	$$-$$0.198$${}^{*}$$
	(0.128)	(0.093)	(0.133)	(0.129)	(0.105)	(0.106)
Unemployment	$-$0.013	0.130	0.085	0.102	0.068	0.085
	(0.114)	(0.119)	(0.120)	(0.116)	(0.108)	(0.107)
State FEs	Yes	Yes	Yes	Yes	Yes	Yes
Clustered SEs (state)	Yes	Yes	Yes	Yes	Yes	Yes
Observations	299	299	299	299	299	299
Adjusted R${}^{2}$	0.819	0.823	0.811	0.813	0.833	0.835

*Note:* $${}^{*}$$ $$p<0.1$$; $${}^{**}$$ $$p<0.05$$; $${}^{***}$$ $$p<0.01$$

The models in Table 2 repeat this analysis with the *AFD* vote share as the dependent variable. Here, we see the opposite results. The *AFD* vote share is inversely related to esoteric beliefs, with the three indicators obtaining negative coefficients throughout. In the combined Model 6, increasing the healer rate from the 5th to the 95th percentile is associated with a decrease of the *AFD* vote share by about 2 percentage points, with a similar decrease for the doctors rate. The presence of a Waldorf school in a district is related to a drop in the *AFD* vote share by about one percentage point. Clearly, these results show that the *AFD* picked up a very different type of sentiment against Corona measures compared to *dieBasis*, and in addition, with much more success. Still, it may be the case that not the absolute level but rather the *change* in the *AFD* vote share from the previous election in 2017 could be affected by esoteric beliefs. However, repeating the above analysis with the difference (2021–2017) in the *AFD* vote share, the results in the Appendix show that there is no evidence for this.

Interestingly, comparing the results for the two parties, we can see differences in how socio-demographic status (proxied by the average income and the level of unemployment) affects the voting results. *dieBasis* achieved higher results in districts with lower levels of unemployment, while the *AFD* performs better in less wealthy districts. This suggests that the support for *dieBasis* does not seem to come from more precarious segments of the population. We also see that urbanization typically goes along with less support for the two parties. For the *AFD*, this finding is in line with much existing research about radical right parties (see for example Harteveld et al. 2022), and similar dynamics seem to be driving the results for *dieBasis*.

### Municipality Level

Do the effects found above persist in the analysis at the municipality level? In the following, the regression analysis is repeated using the sample of municipalities. Due to the fact that municipalities are much smaller, there are much fewer healers, doctors or schools in each of them. Therefore, to ease interpretation, the models below include binary indicators (0/1) for the presence of each, rather than the per capita rate as in the models above. In addition, the models control for the population of each municipality as well as the average household income and the level of unemployment (for the year 2020). Urbanization is measured as the distance to the nearest city, using the dataset described above. The models include district level fixed effects and clustered standard errors. Table 3 shows the results using vote shares for *dieBasis* as the dependent variable.

**Table 3:** OLS regression results at the municipality level. Dependent variable: *dieBasis* vote share. Standard errors clustered at the district level

	*Dependent variable:*					
	*dieBasis* vote share					
	(1)	(2)	(3)	(4)	(5)	(6)
Healer (0/1)	0.063$${}^{**}$$				0.059$${}^{**}$$	0.052$${}^{*}$$
	(0.022)				(0.022)	(0.022)
Doctor (0/1)		0.120$${}^{***}$$			0.103$${}^{***}$$	0.108$${}^{***}$$
		(0.028)			(0.027)	(0.028)
School (0/1)			0.334$${}^{***}$$		0.308$${}^{***}$$	
			(0.063)		(0.062)	
Dist. school (log)				$$-$$0.298$${}^{***}$$		$$-$$0.290$${}^{***}$$
				(0.061)		(0.061)
Population (log)	$$-$$0.113$${}^{*}$$	$$-$$0.110$${}^{*}$$	$$-$$0.103$${}^{*}$$	$$-$$0.129$${}^{**}$$	$$-$$0.158$${}^{**}$$	$$-$$0.182$${}^{***}$$
	(0.044)	(0.046)	(0.047)	(0.044)	(0.047)	(0.045)
Dist. city (log)	0.061	0.066	0.068	0.186$${}^{**}$$	0.070	0.186$${}^{**}$$
	(0.069)	(0.068)	(0.068)	(0.067)	(0.067)	(0.066)
Income	0.007$${}^{*}$$	0.007$${}^{*}$$	0.007$${}^{**}$$	0.005	0.007$${}^{*}$$	0.005
	(0.003)	(0.003)	(0.003)	(0.003)	(0.003)	(0.003)
Unemployment	$$-$$5.671$${}^{**}$$	$$-$$6.118$${}^{**}$$	$$-$$6.148$${}^{**}$$	$$-$$5.520$${}^{**}$$	$$-$$6.340$${}^{**}$$	$$-$$5.772$${}^{**}$$
	(2.021)	(2.027)	(2.022)	(2.022)	(2.032)	(2.035)
District FEs	Yes	Yes	Yes	Yes	Yes	Yes
Clustered SEs (district)	Yes	Yes	Yes	Yes	Yes	Yes
Observations	6,048	6,048	6,048	6,048	6,048	6,048
Adjusted R${}^{2}$	0.333	0.334	0.335	0.341	0.337	0.343

*Note:* $${}^{*}$$ $$p<0.05$$; $${}^{**}$$ $$p<0.01$$; $${}^{***}$$ $$p<0.001$$

Counter to the result above, Model 1 shows a positive effect of healers on the vote share for *dieBasis*, but the effect is small – ceteris paribus, a healer in a municipality is related to an increased vote share by 0.06 percentage points. This effect is higher for doctors (Model 2), with a predicted increase of 0.12 percentage points. Having a school in a municipality is related to the largest increase in electoral support for *dieBasis*, by 0.33 percentage points (Model 3). Given the nationwide election result for *dieBasis* of 1.4%, this is sizable effect. Model 4 again uses an alternative specification for the schools indicator, by including the (log-transformed) minimum distance to the nearest Steiner school. This variable receives a negative coefficient, which indicates that support for dieBasis decreases the further we move away from a school. Models 5 and 6 in Table 3 test all indicators in a single model, which shows that the direction and magnitude of the effects remains largely unchanged in the presence of the other indicators.

**Table 4:** OLS regression results at the municipality level. Dependent variable: *AFD* vote share. Standard errors clustered at the district level

	*Dependent variable:*					
	*AFD* vote share					
	(1)	(2)	(3)	(4)	(5)	(6)
Healer (0/1)	$-$0.199${}^{*}$				$-$0.187${}^{*}$	$-$0.164${}^{*}$
	(0.077)				(0.077)	(0.077)
Doctor (0/1)		$$-$$0.411$${}^{***}$$			$$-$$0.390$${}^{***}$$	$$-$$0.374$${}^{***}$$
		(0.103)			(0.101)	(0.101)
School (0/1)			$-$0.352		$-$0.255	
			(0.210)		(0.207)	
Dist. school (log)				0.905$${}^{***}$$		0.879$${}^{***}$$
				(0.252)		(0.253)
Population (log)	$$-$$0.819$${}^{***}$$	$$-$$0.821$${}^{***}$$	$$-$$0.898$${}^{***}$$	$$-$$0.775$${}^{***}$$	$$-$$0.710$${}^{**}$$	$$-$$0.600$${}^{**}$$
	(0.204)	(0.211)	(0.207)	(0.205)	(0.214)	(0.212)
Dist. city (log)	1.060$${}^{**}$$	1.040$${}^{**}$$	1.048$${}^{**}$$	0.678	1.041$${}^{**}$$	0.679
	(0.365)	(0.363)	(0.366)	(0.360)	(0.363)	(0.357)
Income	$$-$$0.130$${}^{***}$$	$$-$$0.130$${}^{***}$$	$$-$$0.131$${}^{***}$$	$$-$$0.126$${}^{***}$$	$$-$$0.129$${}^{***}$$	$$-$$0.124$${}^{***}$$
	(0.015)	(0.015)	(0.015)	(0.014)	(0.015)	(0.014)
Unemployment	16.213	17.714	16.905	15.764	17.680	16.656
	(10.871)	(10.873)	(10.907)	(11.128)	(10.853)	(11.074)
District FEs	Yes	Yes	Yes	Yes	Yes	Yes
Clustered SEs (district)	Yes	Yes	Yes	Yes	Yes	Yes
Observations	6,048	6,048	6,048	6,048	6,048	6,048
Adjusted R${}^{2}$	0.881	0.881	0.881	0.882	0.881	0.882

*Note:* $${}^{*}$$ $$p<0.05$$; $${}^{**}$$ $$p<0.01$$; $${}^{***}$$ $$p<0.001$$

In Table 4 I repeat the analysis with the AFD vote shares. We find a similar pattern as in the district-level analysis above. If anything, the indicators for esoteric beliefs are inversely related to the AFD vote share, at least for doctors and healers. We find no effect of the presence of Steiner schools on the AFD vote share.

### Municipality Level with Matching

**Fig. 3:**
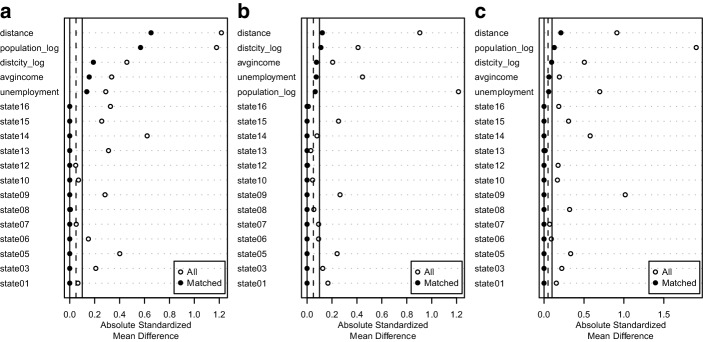
Balance plots for the healers (**a**), doctors (**b**) and schools treatment (**c**)

In a final step, to improve causal identification, genetic matching was used to pre-process the data (Diamond and Sekhon 2013). Three matched samples were generated, using the presence of healers, doctors or schools as treatment, respectively. Matched pairs of treated and control units were created by matching on the control variables in the above analysis: urbanization (distance to the nearest city), average household income and unemployment rate. In addition, matches were restricted to the same state. Fig. 3 shows the balance plots for the three samples.^4^ Overall, matching greatly improved the balance (reduces the distance) between treated and control units, as we can see from the first line in the three plots. If we use the commonly accepted threshold of an absolute standardized mean difference of 0.1 in the covariates, the matched samples for doctors and schools are close to that threshold and can, therefore, be considered to be balanced. This is not the case for the healers sample, which remains unbalanced even after matching due to differences in the population variable. Therefore, results based on the latter sample should be interpreted with a lot of caution.

Using the three matched samples, we estimate the same regression models as above; the results are presented in Table 5. Models 1–3 use the *dieBasis* vote share as the dependent variable. They confirm the positive effects found above. The coefficients indicate that vote shares for *dieBasis* are about 0.09 percentage points higher if a municipality has a homeopathic doctor, and about 0.31 percentage points higher if a Steiner school is located in a municipality. This difference is considerable, given that the average vote share of the party across Germany is only 1.4%. The results for the AFD vote share are similar to the unmatched sample. There is no evidence that schools are related to the AFD vote share, while the negative effect of homeopathic doctors persists.

**Table 5:** OLS regression results at the municipality level on the matched samples. Dependent variables: *dieBasis* and *AFD* vote share. District-level fixed effects and clustered standard errors

	*Dependent variable:*					
	*dieBasis* vote share			*AFD* vote share		
	(1)	(2)	(3)	(4)	(5)	(6)
Healer (0/1)	0.078$${}^{***}$$			$$-$$0.137		
	(0.023)			(0.077)		
Doctor (0/1)		0.085$${}^{*}$$			$$-$$0.317$${}^{**}$$	
		(0.033)			(0.117)	
School (0/1)			0.314$${}^{***}$$			$$-$$0.281
			(0.060)			(0.236)
Population (log)	$$-$$0.152$${}^{**}$$	$$-$$0.175$${}^{***}$$	$$-$$1.003$${}^{**}$$	$$-$$0.889$${}^{***}$$	$$-$$0.949$${}^{**}$$	$$-$$1.049$${}^{*}$$
	(0.055)	(0.045)	(0.324)	(0.239)	(0.308)	(0.469)
Dist. city (log)	0.093	0.053	$$-$$0.247	1.030$${}^{**}$$	0.650$${}^{*}$$	0.860
	(0.070)	(0.048)	(0.165)	(0.391)	(0.314)	(0.552)
Income	0.006$${}^{*}$$	0.006$${}^{*}$$	$$-$$0.015	$$-$$0.133$${}^{***}$$	$$-$$0.135$${}^{***}$$	$$-$$0.157$${}^{***}$$
	(0.003)	(0.003)	(0.010)	(0.018)	(0.017)	(0.028)
Unemployment	$-$2.739	$-$1.582	$-$2.457	18.043	13.302	32.102${}^{*}$
	(1.981)	(1.633)	(3.001)	(13.980)	(14.406)	(14.490)
District FEs	Yes	Yes	Yes	Yes	Yes	Yes
Clustered SEs (district)	Yes	Yes	Yes	Yes	Yes	Yes
Observations	3,484	1,380	242	3,484	1,380	242
Adjusted R${}^{2}$	0.387	0.478	0.489	0.794	0.895	0.747

*Note:* $${}^{*}$$ $$p<0.05$$; $${}^{**}$$ $$p<0.01$$; $${}^{***}$$ $$p<0.001$$

While the result for natural healers has to be treated with caution due to insufficient balance, these results provide robust evidence that electoral support for *dieBasis* in the 2021 election was associated with esoteric beliefs. Due to the setup of the analysis (exact matching on state, district fixed effects), these results are not due to differences between federal states.

## Discussion

Governmental measures such as mask mandates (Huang et al. 2022) or vaccination campaigns (Haas et al. 2021) have proven to be effective in limiting the spread and the impact of the Corona virus. Yet, in many countries, they have been met with considerable resistance in the population. This article shows that this resistance is partly linked to esoteric beliefs, which have a long history and are widespread in Germany and beyond. Usually, these esoteric beliefs were considered to be relevant only for individual choices such as medical treatment; however, during the Corona pandemic, it became apparent that these individual choices can have major societal implications. Using fine-grained election data for Germany, the analysis demonstrates that electoral support for a new party against governmental Corona measures is systematically linked to the presence of homeopathic doctors and Steiner schools in Germany. While this link has often been discussed controversially, this is the first systematic study so far that tests this claim using a comparative analysis that goes beyond narrow samples.

While the results clearly indicate a relationship, the overall magnitude of Corona-related criticism that can be attributed to esoteric beliefs remains modest, since the overall voting result for the *dieBasis* party is low at the national level. The right-wing *Alternative für Deutschland* (AFD) that also ran against Corona measures absorbed much of these tendencies originating from the political right, with a much higher result the national level. However, this type of opposition follows a different pattern, since the analysis finds no evidence that the AFD benefited systematically from these beliefs.

## Appendix

### District-level Analysis: Difference in AFD Vote Shares (2021–2017)

**Table 6:** OLS regression results at the electoral district level. Dependent variable: difference in *AFD* vote share (2021–2017). Standard errors clustered at the state level

	*Dependent variable:*					
	Difference *AFD* vote share (2021–2017)					
	(1)	(2)	(3)	(4)	(5)	(6)
Healer rate	$-$1.252				$-$1.168	$-$1.267
	(1.411)				(1.282)	(1.241)
Doctor rate		$-$1.842			$-$0.898	$-$1.205
		(4.650)			(4.829)	(4.775)
School rate			$-$2.849		1.300	
			(20.759)		(20.057)	
School (0/1)				0.053		0.082
				(0.145)		(0.137)
Urban	$$-$$0.582$${}^{***}$$	$$-$$0.584$${}^{***}$$	$$-$$0.592$${}^{***}$$	$$-$$0.611$${}^{***}$$	$$-$$0.578$${}^{***}$$	$$-$$0.597$${}^{***}$$
	(0.179)	(0.179)	(0.169)	(0.164)	(0.172)	(0.167)
Income	$$-$$0.098$${}^{***}$$	$$-$$0.099$${}^{***}$$	$$-$$0.102$${}^{***}$$	$$-$$0.105$${}^{***}$$	$$-$$0.096$${}^{***}$$	$$-$$0.099$${}^{***}$$
	(0.019)	(0.025)	(0.020)	(0.020)	(0.023)	(0.023)
Unemployment	$$-$$0.268$${}^{***}$$	$$-$$0.261$${}^{***}$$	$$-$$0.262$${}^{***}$$	$$-$$0.264$${}^{***}$$	$$-$$0.267$${}^{***}$$	$$-$$0.269$${}^{***}$$
	(0.042)	(0.040)	(0.039)	(0.038)	(0.044)	(0.043)
State FEs	Yes	Yes	Yes	Yes	Yes	Yes
Clustered SEs (state)	Yes	Yes	Yes	Yes	Yes	Yes
Observations	299	299	299	299	299	299
Adjusted R${}^{2}$	0.596	0.595	0.595	0.595	0.593	0.594

*Note:* $${}^{*}$$ $$p<0.1$$; $${}^{**}$$ $$p<0.05$$; $${}^{***}$$ $$p<0.01$$

## Footnotes

1 https://www.bdh-online.de/patienten/therapeutensuche/.

2 https://www.homoeopathie-online.info/arztsuche/.

3 https://www.waldorfschule.de/schulen/schulsuche/schulverzeichnisse.

4 The *state* variables are perfectly balanced by design, due to the requirement of perfect matching on the state ID.
